# Evaluation of the test–retest and inter-mode comparability of the Impact of Vision Impairment questionnaire in people with chronic eye diseases

**DOI:** 10.1007/s00417-023-06334-4

**Published:** 2024-01-05

**Authors:** Jan Henrik Terheyden, Reglind A. D. Ost, Charlotte Behning, Liza Mekschrat, Gamze Bildik, Maximilian W. M. Wintergerst, Frank G. Holz, Robert P. Finger

**Affiliations:** 1 Department of Ophthalmology, University Hospital Bonn, Ernst-Abbe-Str. 2, 53127 Bonn, Germany; 2 Department of Medical Biometry, Informatics and Epidemiology, University Hospital Bonn, Bonn, Germany; 3 Department of Ophthalmology, University Hospital Mannheim, University of Heidelberg, Mannheim, Germany

**Keywords:** Patient-reported outcome, Reliability, Impact of Vision Impairment scale, Quality of life

## Abstract

**Purpose:**

The main objective of this study is to assess the test–retest and inter-administration mode reliability of the Impact of Vision Impairment profile (IVI), a common patient-reported outcome measure (PROM) for people with chronic eye diseases.

**Methods:**

The IVI was administered to adult patients with stable, chronic eye diseases two to four times per participant (average intervals between administrations 12 to 20 days; maximum two phone interviews, paper administration, electronic administration) by two trained interviewers. Rasch models were fit to the data. Intra-class correlation coefficients (ICCs), mean differences and Cronbach’s alpha between test–retest administrations (two phone interviews) and inter-mode comparisons were calculated.

**Results:**

Two hundred-sixteen patients (mean age 67 ± 12 years, 40% male) were included in the study. The IVI met all psychometric requirements of the Rasch model, and the division into the domains of functional items (IVI_F) and emotional items (IVI_E) corresponded to the German validation study. ICCs (all for IVI_F and IVI_E, respectively) for the retest administrations were 0.938 and 0.912, and 0.853 and 0.893 for inter-mode comparisons phone/paper, 0.939 and 0.930 for phone/electronic, and 0.937 and 0.920 for paper/electronic (all *p* < 0.01). Mean differences (all for IVI_F and IVI_E, respectively) for the retest administrations were 2.8% and 0.7% and ranged from 2.0% to 6.2% and from 0.4 % to 4.9% between administration modes. Cronbach’s alpha ranged from 0.886 to 0.944 for retest and inter-mode comparisons.

**Conclusion:**

Due to the high test–retest reliability and the almost equally high comparability of different modes of administration of the IVI, the study endorses its use as a robust PROM to capture vision-related quality of life. Our results further support the use of the IVI as an endpoint in clinical trials and may simplify implementing it in both clinical trials or real-world evidence generation by offering multiple administration modes with high reliability.

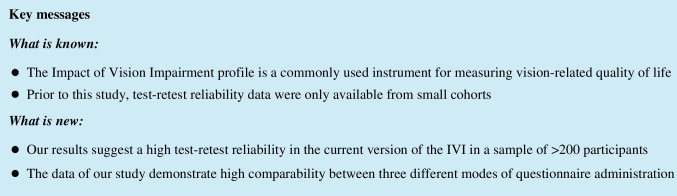

**Supplementary Information:**

The online version contains supplementary material available at 10.1007/s00417-023-06334-4.

## Introduction

Patient-reported outcome measures (PROMs) continue to gain importance in both routine eye care provision and research [1–4]. PROMs consist of several questions assessing the patient’s health from their perspective [4]. One of the most common concepts measured by PROMs in ophthalmology is vision-related quality of life (VRQoL). It is known to be compromised by even mild forms of visual impairment and is therefore considered as a useful tool to gain additional information to visual function testing [5–7]. Typical PROMs in ophthalmology to assess VRQoL or visual functioning are the Impact of Vision Impairment (IVI) profile, the National Eye Institute Visual Functioning Questionnaire-25 (NEI-VFQ-25), and the Visual Function Index-14 (VF14) [4, 8, 9]. Similar to psychophysical function tests, PROM instruments require careful validation [10, 11]. One distinguishes between qualitative validation steps, where, e.g., content validity is assessed during the development phase of a questionnaire and quantitative validation steps [12]. The latter include an assessment of reliability and, e.g., construct validity of a questionnaire by statistical techniques such as exploratory factor analysis, to measure if the questions measure the construct they are intended to measure [12]. Similarly, reliability is assessed using quantitative metrics such as Cronbach’s alpha or Rasch model person reliability to assess the internal consistency of the scale, and methods comparing two assessments with the same patient at different time points to examine the test–retest reliability of the scale [12].

Both the NEI-VFQ-25 and the VF14 have undergone test–retest reliability testing in large cohorts (NEI-VFQ-25 *n* = 186; VF-14 *n* = 383, which showed a high agreement between repeated administrations of both instruments [13, 14]. The IVI is widely used in rehabilitation and treatment studies [9, 11, 15–20], but to date, data on test–retest reliability have been generated in three studies that have included comparatively small subgroups of participants (*n* ≤ 60 participants per study, total 102 test–retest participants in all available studies) [21–23]. Regarding this limited evidence, we identified the need to reevaluate test–retest reliability in a larger cohort.

Another important aspect of questionnaire studies besides repeatability over different time points is the repeatability across different administration modes. Many questionnaire studies are still conducted with self-administered paper questionnaires or with interviews. However, due to numerous advantages of electronic modes of administration, such as higher and faster response rates, lower costs, and simplified data analysis, these have become more popular in research [24]. To our knowledge, a systematic comparison of electronic, paper-based, and interviewer-based modes has not been conducted in any of the studies mentioned above. One single study has compared self-administration of the IVI using a paper form and interviewer administration in a cohort with 31 participants, which is a similarly small sample size compared to the test–retest reliability studies described [23].

To fill this gap, our objective was to investigate the test–retest reliability and inter-mode comparability of the IVI concerning paper, phone, and electronic administration modes, hypothesizing that retest assessments are comparable but interviewer administration yielding higher IVI scores [25, 26].

## Methods

### Recruitment

**Fig. 1:**
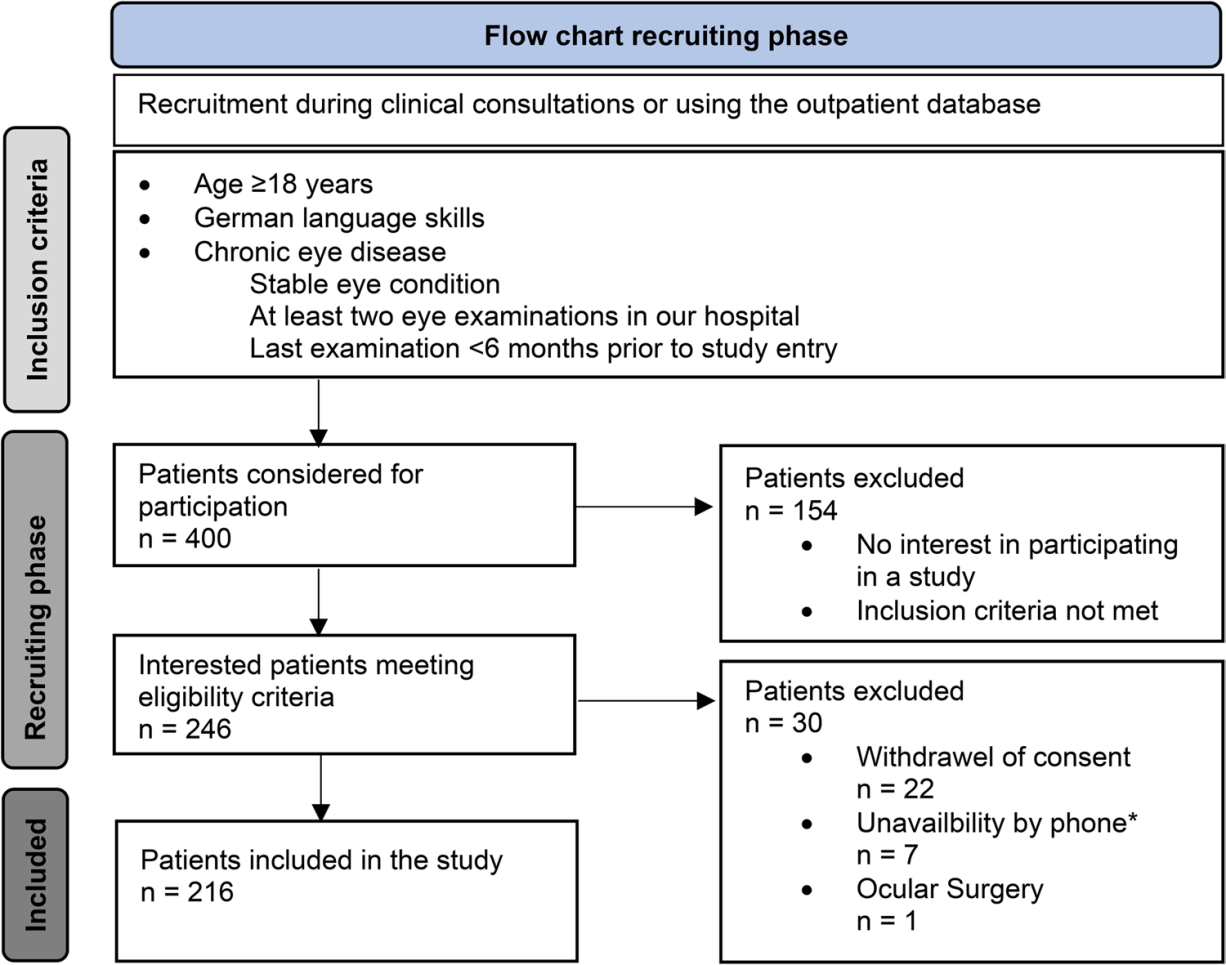
Flow chart of patient recruitment. *At least five phone call attempts

Our prospective study was carried out at the Department of Ophthalmology, University Hospital Bonn, Germany, from April 2020 to December 2020. Ethical approval was obtained from the Institutional Review Board of the University Hospital Bonn (reference number: 130/16). Patients were recruited during clinical consultations and from an outpatient database. Inclusion criteria were chronic eye conditions, age ≥ 18 years and sufficient German language skills. We only included individuals with stable chronic eye condition for at least two previous examinations at our hospital, the latter being no more than 6 months prior to study entry. Exclusion criteria were any acute-onset eye diseases < 6 weeks, any eye surgery or interventions during or < 6 weeks prior to study participation except intravitreal injections. The size of the sample was chosen according to international recommendations [27]. Written informed consent was obtained from all participants of the study. The study protocol followed the principles of the Declaration of Helsinki (Fig. 1).

### The Impact of Vision Impairment questionnaire

The IVI questionnaire consists of 28 items to detect different aspects of VRQoL in patients with visual impairment [28]. It was originally developed to evaluate whether patients are limited in their daily lives due to reduced vision and may need rehabilitation [29, 30]. As indicated in its validation study, the German IVI includes four response options for items 1–13 and five response options for items 14–28, starting with “not at all” and ending with “very often” to evaluate the items’ influence on VRQoL. The fifth option “don’t do this for other reasons” was treated as missing in our analysis. The questionnaire is divided into two subscales: “Functional IVI” (items 1–20) and “Emotional IVI” (items 21–28) [5]. In accordance with the original IVI, the German version was initially developed for interview administration [5, 29].

### Questionnaire administration

**Fig. 2:**
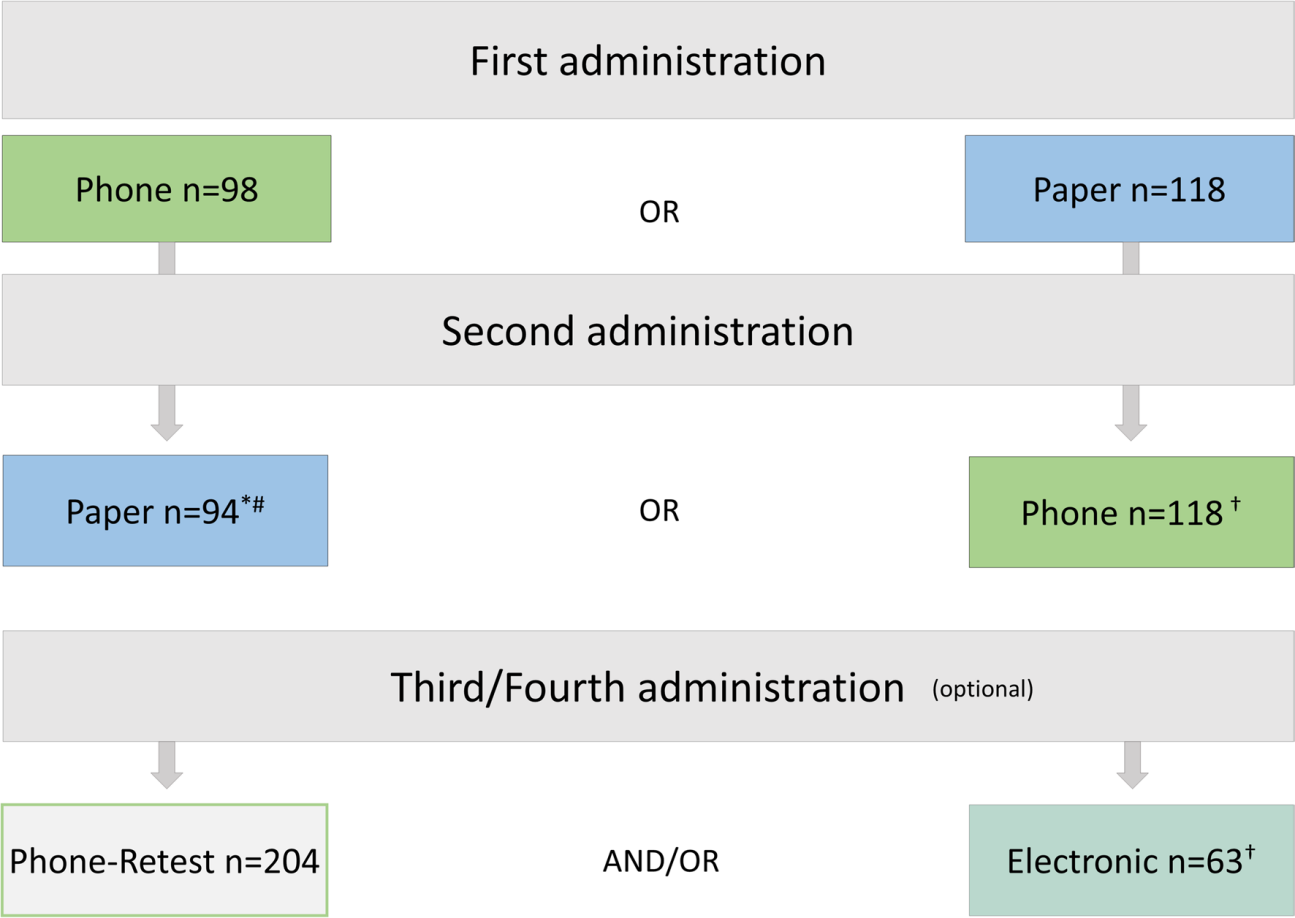
Order of questionnaire administrations. Up to four sets of IVI questionnaire data were collected per participants. Interviewer administrations were performed remotely (phone) and self-administrations were performed using paper or electronic forms. ^*^Omitted in 4 participants due to vision impairment; ^#^Performed as third mode by one participant; ^†^Performed as third mode by two participants (after paper and electronic)

The study included two to four administrations of the IVI for each participant (Fig. 2). The initial mode of administration depended on the participant’s preference. The required response interval between administrations was ≤ 10 weeks. During the repeated administrations, the previous questionnaire responses were unavailable to both the interviewer and the participant. The interviewer administrations were conducted by two trained interviewers who instructed the participants according to recommended PROM administration guidelines [31].

In addition to the administration of the IVI, participants self-administered structured questionnaires on socio-demographical data and medical history. Diagnoses and visual acuity data from the last visit were obtained from the patient files. Regarding missing data, the questionnaire was excluded from the study if responses were available for fewer than 50% of items.

### Psychometric validation of the IVI in our population

We performed Rasch analysis, which transforms the ordinal Likert scales into pseudo-interval-level scales (expressed in logits) and thus allows for parametric statistical analysis using commercial software (Winsteps software, ver. 3.92.1.2; Winsteps, Chicago, IL), employing the Andrich Rating Scale model [32, 33]. As an instance of latent trait models, the application of a Rasch model minimizes the impact of single missing responses since the measured construct is largely independent from individual missing values [34]. We anchored the person measures across modes of administration to the Rasch model based on the first interviewer administration to enable direct comparability of the results within our study, as well as in relation to other studies of the IVI [5, 35, 36]. Apart from this, we also analyzed item measures of Rasch models without this anchoring step, by Pearson correlation coefficients. After processing the questionnaire data, we inverted the person measures of the IVI to facilitate interpretation by assigning lower person measures to participants with more severe vision impairment.

Based on the Rasch models, we assessed threshold ordering, person reliability, person separation, item fit, and differential item functioning (DIF). Response category threshold ordering was assessed to verify whether the category structure belonging to the items is matching [28]. A person separation index (PSI) above 2.0 was considered as good and values between 1.5 and 2.0 as acceptable, and a person reliability (PR) above 0.8 was deemed high and between 0.6 and 0.8 deemed acceptable [37–39]. Unidimensionality concerning the underlying construct was assessed using the infit and outfit mean square standardized residuals (MNSQ) statistic, with values up to 1.4 being reasonable for a rating scale survey [40]. The targeting of the instrument was identified by calculating the difference between person and item means, with values > 1.0 logits representing mistargeting. Lastly, differential item functioning (DIF) was assessed for each item to measure differences between subgroups concerning sex and age and was noticeable for values over 0.64 [41]. To determine the significance of deviating DIF values, a comparison of the person measures with and without retention of the conspicuous values was performed using intra-class correlation coefficients (ICCs).

### Statistical analysis

The main outcomes of our study were ICCs and mean differences of repeated administration. We investigated (1) test–retest and (2) inter-mode reliability using two-way mixed effect ICCs with absolute agreement as ICCs are the preferred assessment for settings where measures are repeated [42–44]. ICCs were interpreted according to the categories suggested by Cicchetti and Sparrow (poor ICC < 0.40; fair, 0.40 ≤ ICC ≤ 0.59; good, 0.60 ≤ ICC ≤ 0.74; excellent, 0.75 ≤ ICC ≤ 1.00) [45]. Furthermore, we displayed the mean of inter-measurement differences ± 1.96 standard deviations as limits of agreement in Bland–Altman plots [46]. The coefficient of repeatability (CoR, i.e., an indicator of absolute reliability) was calculated as 1.96 standard deviations of the mean test–retest difference [47, 48]. Cronbach’s alpha, a conventional test theory metric of reliability, was also calculated for the test–retest reliability assessment, based on person measures [12]. Deming regression analysis was performed to compare person measures between different administrations [49]. In contrast to classical linear regression, Deming regression considers the errors in both variables included in the respective regression model [50]. Intercept and slope were evaluated for significant deviations from 0 and 1, respectively [49]. To rule out any systematic bias, we compared the distribution of personal characteristics of participants who selected interviewer assessment and participants who selected self-assessment as the initial mode of administration, using the *t* test and the chi-squared test. In addition, we investigated which influence the initial mode of administration, visual acuity, self-reported hearing difficulties, self-reported psychiatric diseases, and the interval between administrations had on the mean differences between test–retest administration and phone-paper administration, using the Mann–Whitney *U* test.

Statistical analyses were performed with SPSS (Version 27 software, IBM SPSS Inc., Armonk, MY) and R (version 4.1.2). *P* values < 0.05 were considered statistically significant with correcting for multiple testing as necessary.

## Results

Two hundred sixteen out of 246 total participants completed at least two administrations of the IVI and were included in the analysis. The reasons for drop-out were withdrawal of consent (*n* = 22), unavailability of participants by phone (*n* = 7), and ocular surgery (*n* = 1). The majority of participants (160 individuals, 74%) was above 60 years of age (Table 1). The most frequent ocular conditions in our cohort were vitreoretinal diseases, glaucoma and cataract (Supplementary Table 1). A total of 204 individuals were included in the test–retest assessment. The availability of administration modes for the inter-mode assessment was 216 participants (100%) for interviewer administrations, 212 (98%) for self-administrations using pen-and-paper, and 63 (29%) for electronic self-administrations. The mean administration intervals were 18 ± 13 days for the test–retest assessments, 12 ± 12 days between phone and paper administrations, 20 ± 13 days between paper and electronic administrations, and 17 ± 11 days between phone and electronic administrations. The number of individuals who chose self-administration as the initial mode of administration (*n* = 118) was higher than the number of participants who chose to be interviewed first (*n* = 98) but no differences between these groups in terms of socio-demographic characteristics, ocular or systemic concurrent disease were present (all *p* ≥ 0.087, Supplementary Table 1). Questionnaires with at least half of the responses missing were excluded from the study (*n* = 5). The number of single missing values amounted to 1.5%, or 0.01% of all answers, excluding the values of the answer option “Don’t do this for other reasons “, which was considered missing in our analysis.

**Table 1:** Characteristics of the sample

	*n* (%)
	All (*n* = 216)
Age	
Mean age [years] ± SD	67 ± 12
Sex	
Female (%)	129 (59.7)
Male (%)	87 (40.3)
Visual acuity (logMAR)	
Hearing difficulties	0.27 ± 0.14
Yes (%)	59 (27.3)
No (%)	156 (72.2)
Missing (%)	1 (0.4)
Education	
Elementary school (%)	76 (35.2)
Secondary school (%)	78 (36.1)
High school (%)	22 (10.2)
University with graduation (%)	36 (16.7)
Missing (%)	4 (1.9)
Employment status	
Working (%)	56 (25.9)
Unemployed (%)	22 (10.2)
Retired (%)	130 (60.2)
Missing (%)	8 (3.7)
Living situation	
Alone (%)	64 (29.6)
With others (%)	148 (68.5)
Missing (%)	4 (1.9)
Marital status	
Married (%)	127 (58.8)
Widowed (%)	36 (16.7)
Divorced (%)	32 (14.8)
Unmarried (%)	21 (9.7)

### Rasch model fit

All response category thresholds were ordered. Three items demonstrated misfit (infit MNSQ 1.45, item 1; 1.56, item 14; 1.40, item 21), but the values were below a level that degrades the measurement system, and removal did not improve fit statistics, so we retained the items [40]. PR and PSI indicated adequate internal consistency (Table 2). The difference in person and item mean showed poor person-item targeting in our cohort. Three items were indicative of DIF by sex and one item by age group, but person measures were unchanged after removal, and the items could therefore be retained (IVI_F, phone administration, ICC 0.997, 95%-CI [0.996; 0.998]; IVI_F, paper administration, ICC 0.996, 95%-CI [0.995; 0.997]; IVI_E, electronic administration, ICC 0.979, 95%-CI [0.847; 0.993]). The resulting person measures were highest for the interview administrated IVI (Supplementary Table 2). Pearson correlation coefficients between item measures were calculated to validate the chosen anchoring method. They were 0.975 [0.946; 0.988] for the test and retest-phone administrations, 0.878 [0.751; 0.942] for phone and paper administration, and 0.877 [0.748; 0.942] for phone and electronic administration.

**Table 2:** Fit parameters of the phone, paper and electronic administration for the functional and emotional IVI

Parameters	Rasch model	Phone administration		Paper administration		Electronic administration	
		IVI_F	IVI_E	IVI_F	IVI_E	IVI_F	IVI_E
Threshold ordering, *n*	0	0	0	0	0	0	0
Misfitting items, *n*	0	0	**item21**^**a**^	0	0	**item1**^**a**^; **item14**^**a**^	0
PSI	> 2.0 (1.5)	2.55	1.76	3.32	2.71	3.33	2.19
PR	> 0.8 (0.6)	0.87	0.76	0.92	0.88	0.92	0.83
Difference in person and item mean	< 1	**1.91**	**2.60**	**1.56**	**2.87**	**2.38**	**3.97**
DIF	< 0.64						
Age (≤ 60; > 60)		None	None	None	None	None	**item28**^**b**^
Sex (female; male)		**item1**^**b**^	None	**item1**^**b**^**; item9**^**b**^**; item18**^**b**^	None	None	None

*DIF*, Differential item functioning; *IVI_F*, functional subscale of the IVI; *IVI_E*, emotional subscale of the IVI; *PR*, person reliability; *PSI*, person separation index

Bold values represent misfit to the Rasch model

^a^Values are not degrading the measurement system [40]

^b^No influence on measurements (ICC ≥ 0.98)

### Test–retest and inter-mode reliability

**Fig. 3:**
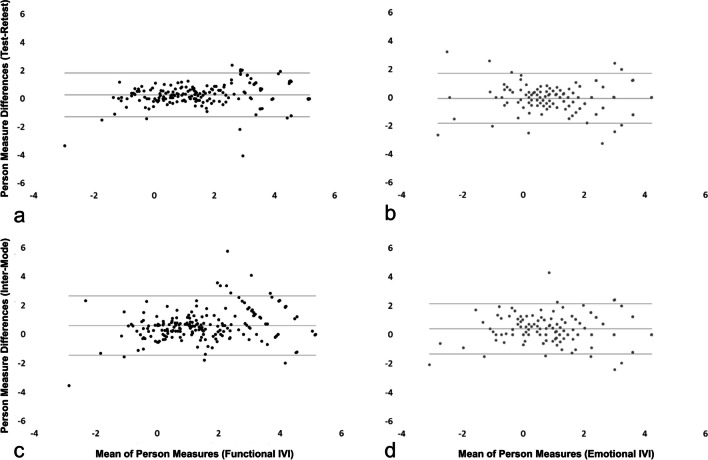
Bland–Altman plots of test–retest assessments (phone administration) of the functional (**a**) and emotional (**b**) subscales and of inter-mode comparability assessments (phone and paper administration) of the functional (**c**) and emotional (**d**) IVI subscales display the distributions of retest differences in person measures (higher vision-related quality of life corresponds to higher person measures)

ICCs were excellent, both for test–retest and inter-mode comparisons (Table 3). Mean differences between test–retest and inter-mode comparisons were comparable, and the Bland–Altman analysis did not indicate any systematic bias (Fig. 3). Deming regression revealed that absolute differences of test–retest assessments were not significantly different from each other when the same mode of administration was used. Phone administration compared to paper and electronic administration resulted in significantly higher functional IVI subscale scores (i.e., higher VRQoL), and paper administration compared to phone and electronic administration yielded significantly lower emotional IVI subscale scores (i.e., lower VRQoL) when comparing the Deming regression intercepts (Table 3; Supplementary Table 2).

**Table 3:** Reliability metrics of the test–retest and inter-mode assessments for the functional and emotional IVI subscales

		Test–retest		Inter-mode	
		*Phone–phone (n* = *204)*	*Phone–paper (n* = *212)*	*Phone–electronic (n* = *63)*	*Paper–electronic (n* = *63)*
IVI_F	ICC (average values)	0.938	0.853	0.939	0.937
	95%-CI	[0.909; 0.957]	[0.693; 0.917]	[0.896; 0.964]	[0.894; 0.962]
	Cronbach’s *α*	0.94	0.89	0.94	0.94
	Mean difference (% subscale range)	0.27 (2.8)	0.61 (6.2)	0.24 (2.5)	− 0.20 (2.0)
	CoR (% subscale range)	1.55 (15.8)	2.06 (21.0)	1.61 (16.5)	1.53 (15.6)
	Deming, intercept	− 0.10	− 0.29	− 0.27	− 0.18
	95%-CI	[− 0.27; 0.07]	[− 0.53; − 0.06]^*^	[− 0.54; − 0.01]^*^	[− 0.43; 0.07]
	Deming, slope	0.91	0.83	1.02	1.24
	95%-CI	[0.82; 0.99]^*^	[0.72; 0.94]^*^	[0.86; 1.17]	[1.06; 1.42]^*^
IVI_E	ICC (average values)	0.912	0.893	0.930	0.920
	95%-CI	[0.884; 0.933]	[0.816; 0.933]	[0.885; 0.958]	[0.866; 0.952]
	Cronbach’s *α*	0.91	0.91	0.93	0.92
	Mean difference (% subscale range)	− 0.06 (0.7)	0.41 (4.9)	0.03 (0.4)	− 0.20 (2.3)
	CoR (% subscale range)	1.77 (21.2)	1.75 (20.9)	1.42 (16.9)	1.40 (16.8)
	Deming, intercept	0.01	− 0.38	0.25	0.35
	95%-CI	[− 0.27; 0.28]	[− 0.60; − 0.16]^*^	[− 0.17; 0.67]	[0.16; 0.54]^*^
	Deming, slope	1.03	0.99	0.83	0.89
	95%-CI	[0.90; 1.16]	[0.88; 1.09]	[0.62; 1.04]	[0.76; 1.03]

*ICC*, Intra-class correlation coefficient; *IVI_F*, functional subscale; *IVI_E*, emotional subscale; *ICC*, intra-class correlation coefficient; *CoR*, coefficient of repeatability

^*^Regression intercept significantly different from 0 or slope significantly different from 1

### Sensitivity analysis

To validate the above findings, we investigated relationships between mean differences of the test–retest or inter-mode comparisons and five potential confounders, correcting for multiple testing with the Bonferroni method. Neither best-corrected visual acuity (corrected *p* ≥ 0.546), nor psychiatric diseases (corrected *p* ≥ 1.0), self-reported hearing difficulties (corrected *p* ≥ 1.0), administration interval (corrected *p* ≥ 1.0), nor the initial mode of administration (corrected *p* ≥ 1.0) were significantly associated with the IVI subscale score differences (Supplementary Table 3).

## Discussion

The purpose of this study was to evaluate test–retest reliability and inter-mode reliability. Test–retest differences were small and non-significant, and inter-mode variations were in a similar, small range. We found a small but significant reduction in VRQoL scores when questionnaires were self-administered using paper forms compared to phone administration. Due to the excellent ICC values (IVI_F 0.853; IVI_E 0.893) and low mean differences (IVI_F 6% of subscale range, IVI_E 5% of subscale range), we classified these deviations in the statistically but not clinically relevant range [45]. Overall, our data demonstrate that the IVI questionnaire is highly reliable, independent of repeated assessment or the mode of administration, also supported by the strong associations of the item measures across different modes. Our results were not affected by the initial mode of administration, participants’ visual or hearing impairment, psychiatric diseases, or the time between IVI administrations.

The test–retest reliability of the IVI was largely comparable to what has been reported previously in smaller cohorts (*n* = 20 to 60) [21–23]. ICCs between IVI test–retest scores in our sample (0.94 for IVI_F and 0.91 for IVI_E) were minimally higher compared to the values reported by the original 32-item IVI by Weih et al. (0.88 for IVI total sum score) and mean differences were lower in our study (≤ 2.8% of subscale range versus 6.1% of scale range) [23]. Test–retest subgroup analyses in the Greek and Thai validations of the IVI reported similar results to our study (ICCs ≥ 0.90, Cronbach’s *α* > 0.75) [21, 22]. Other commonly used ophthalmic PROMs, such as the NEI-VFQ-25 or the VF-14, achieve similarly ICCs (0.57–0.91), which places the IVI in the series of reliable PROMs in ophthalmology [13, 14, 51].

The implementation of different modes of administration did not have a relevantly higher impact on the IVI subscale scores than performing a retest administration in our study. As indicated above, the inter-mode reliability has only been investigated in one study (*n* = 31) that compared self-administration to interview-administration, using IVI-32 sum scores which are no longer recommended to be used [23]. In contrast to Weih et al. we have investigated the inter-mode reliability of the IVI using more state-of-the-art statistical approaches including Rasch-based person measures. Our results suggested a higher agreement between modes of administration than previously reported (mean differences ≤ 6.2% in this study versus ≤ 9.5% in the previous analysis) [23]. Additionally, our findings of the electronic questionnaire with excellent ICCs ≥ 0.920 further support the implementation of electronic PROMs in ophthalmology (Table 3) [24].

We found a trend of higher reported vision-related quality of life in interviews compared to pen and paper assessments, which is known from other PROMs such as the vision core measure (VCM1) study by Frost et al. [25]. In our dataset, mean differences between different modes of administration were small (≤ 6.2% of functional subscale range; ≤ 4.9% of emotional subscale range), further supporting the equivalence between the investigated administration modes for the IVI. When comparing paper to electronic self-administration, mean differences were ≤ 2.3% of the respective subscale range. This is in line with previous research, which has identified 5% of the scale range as the cutoff value for most studies comparing paper to electronic administrations of various PROMs [52]. Only few other studies have systematically compared the use of different modes of administration in ophthalmic PROMs, and reported inter-mode reliabilities were in an overall similar range to the results of our study [53, 54].

The main limitation of our study is the targeting of the sample which is a result of including individuals with several chronic eye diseases but not only visually impaired individuals [55]. The problem of inadequate person-item targeting is known from previous studies of the IVI [56]. We did not randomize participants by initial mode of administration since most participants asked to start with pen and paper administration. Yet, a post hoc analysis did not reveal any differences based on the initial mode of administration. An additional aspect not investigated in our study is how the participants’ health literacy may modify the reliability of the IVI, which should be further investigated in future studies [57].

Our study’s main strengths include its large sample size in which both test–retest and inter-mode reliability of the IVI profile have been tested, the heterogeneity of the sample making the results more likely to be generalizable to a population accessing eye care services, and the use of latent trait models which have several known advantages over sum scoring in psychometric assessment [58, 59].

In conclusion, the IVI questionnaire demonstrated excellent test–retest reliability and our data suggest the use of interview, paper-based, or electronic modes of administration to be comparable in our study cohort. The results may facilitate implementing the IVI in clinical routine and research because of a reduced administration burden.

### Supplementary Information

Below is the link to the electronic supplementary material.

Supplementary file1 (DOCX 645 KB)

## Data Availability

The datasets used and analyzed during the current study are available from the corresponding author on reasonable request.

## Abbreviations

*CoR*: Coefficient of repeatability
*DIF*: Differential item functioning
*ICC*: Intra-class correlation coefficient
*IVI*: Impact of Vision Impairment profile
*IVI_F*: Functional subscale of the Impact of Vision Impairment profile
*IVI_E*: Emotional subscale of the Impact of Vision Impairment profile
*MNSQ*: Mean square standardized residuals
*PR*: Person reliability
*PROMs*: Patient-reported outcome measures
*PSI*: Person separation index
*VRQoL*: Vision-related quality of life
