# Supplementary material for: Evaluation of the test–retest and inter-mode comparability of the Impact of Vision Impairment questionnaire in people with chronic eye diseases
Source: Graefes Arch Clin Exp Ophthalmol. 2024 Jan 5;262(6):1933–43. doi: 10.1007/s00417-023-06334-4 (PMC11106107; doi:10.1007/s00417-023-06334-4)

# Appendices

| **Supplementary Table 1.** Characteristics of the sample sorted by the first administration mode | | | | | |  |
| --- | --- | --- | --- | --- | --- | --- |
|  | | **n (%)** |  |  |  |  |
|  | | **All (n=216)** |  | **phone first (n=98)** | **paper first** |  |
|  |  |  |  |  | **(n=118)** | **p-value^c^** |
| **Age** | |  |  |  |  | 0.169 |
| ≤60 | | 56 (25.93) |  | 21 (21.43) | 35 (29.66) |  |
| >60 | | 160 (74.07) |  | 77 (78.57) | 83 (70.34) |  |
| Mean age (in years) | | 66.65 |  | 67.86 | 65.66 | 0.158 |
|  | |  |  |  |  |  |
| **Sex** | |  |  |  |  | 0.491 |
| Female | | 129 (59.72) |  | 61 (62.24) | 68 (57.63) |  |
| Male | | 87 (40.27) |  | 37(37.76) | 50 (42.37) |  |
|  | |  |  |  |  |  |
| **Visual impairment (better eye)** | |  |  |  |  | 0.634 |
| absent (≤ 0.3 logMAR) | | 153 (70.83) |  | 71 (72.45) | 82 (69.49) |  |
| present (>0.3 logMAR) | | 63 (29.17) |  | 27 (27.56) | 36 (30.51) |  |
|  | |  |  |  |  |  |
| **Eye conditions (better eye)^d^** | |  |  |  |  |  |
| Glaucoma | | 46 (21.30) |  | 22 (22.45) | 24 (20.34) | 0.706 |
| Cataract | | 43 (19.91) |  | 19 (19.39) | 24 (20.34) | 0.862 |
| Age-related macular degeneration | | 37 (17.13) |  | 19 (19.39) | 18 (15.25) | 0.422 |
| Ocular surface disease | | 34 (15.74) |  | 17 (17.35) | 17 (14.41) | 0.555 |
| Diabetic retinopathy | | 18 (8.33) |  | 7 (7.14) | 11 (9.32) | 0.564 |
| Myopic macular degeneration | | 21 (9.72) |  | 10 (10.20) | 11 (9.32) | 0.828 |
| Retinal detachments | | 14 (6.48) |  | 4 (4.08) | 10 (8.47) | 0.192 |
| Macular pucker | | 11(5.09) |  | 3 (3.06) | 8 (6.78) | 0.216 |
| Other vitreoretinal diseases | | 48 (22.22) |  | 18 (18.37) | 30 (25.42) | 0.214 |
|  | |  |  |  |  |  |
|  | |  |  |  |  |  |
| **Nonocular comorbidities^d^** | |  |  |  |  |  |
| Systemic arterial hypertension | | 96 (44.44) |  | 39 (39.80) | 57 (48.31) | 0.210 |
| None | | 54 (25.00) |  | 25 (25.51) | 29 (24.58) | 0.875 |
| Other chronic diseases | | 49 (22.69) |  | 24 (24.49) | 25 (21.19) | 0.564 |
| Diabetes mellitus | | 44 (20.37) |  | 19 (19.39) | 25 (21.19) | 0.744 |
| Musculoskeletal diseases | | 44 (20.37) |  | 25 (25.51) | 19 (16.10) | 0.087 |
| Depression and other mental illness | | 32 (14.81) |  | 13 (13.27) | 19 (16.10) | 0.559 |
| Cardiovascular diseases | | 35 (16.20) |  | 16 (16.33) | 19 (16.10) | 0.964 |
| Pulmonary diseases | | 30 (13.89) |  | 14 (14.29) | 16 (13.56) | 0.878 |
| Neoplastic diseases | | 20 (9.26) |  | 11 (11.22) | 9 (7.63) | 0.364 |
| Neurological disorders | | 15 (6.94) |  | 7 (7.14) | 8 (6.78) | 0.917 |
|  | |  |  |  |  |  |
| **Hearing difficulties** | |  |  |  |  | 0.784 |
| Yes | | 59 (27.31) |  | 26 (26.53) | 33 (27.97) |  |
| No | | 156 (72.22) |  | 72 (73.47) | 84 (71.19) |  |
| Missing | | 1 (0.46) |  | 0 (0.00) | 1 (0.85) |  |
|  | |  |  |  |  |  |
| **Education** | |  |  |  |  | 0.245 |
| Elementary school | | 76 (35.19) |  | 42 (42.86) | 34 (28.81) |  |
| Secondary school | | 78 (36.11) |  | 30 (30.61) | 48 (40.68) |  |
| High school | | 22 (10.19) |  | 10 (10.20) | 12 (10.17) |  |
| University with graduation | | 36 (16.67) |  | 14 (14.29) | 22 (18.64) |  |
| Missing | | 4 (1.85) |  | 2 (2.04) | 2 (1.69) |  |
|  | |  |  |  |  |  |
| **Employment status** | |  |  |  |  | 0.119 |
| Working | | 56 (25.92) |  | 21 (21.43) | 35 (29.66) |  |
| Unemployed | | 22 (10.19) |  | 7 (7.14) | 15 (12.71) |  |
| Retired | | 130 (60.19) |  | 67 (68.37) | 63 (53.39) |  |
| Missing | | 8 (3.70) |  | 3 (3.06) | 5 (4.24) |  |
|  | |  |  |  |  |  |
| **Living situation** | |  |  |  |  | 0.659 |
| Alone | | 64 (29.63) |  | 29 (29.59) | 35 (29.66) |  |
| With others | | 148 (68.52) |  | 67 (68.37) | 81 (68.64) |  |
| Missing | | 4 (1.85) |  | 2 (2.04) | 2 (1.70) |  |
|  | |  |  |  |  |  |
| **Marital status** | |  |  |  |  | 0.140 |
| Married | | 127 (58.80) |  | 58 (59.18) | 69 (58.47) |  |
| Widowed | | 36 (16.67) |  | 20 (20.41) | 16 (13.56) |  |
| Divorced | | 32 (14.81) |  | 15 (15.31) | 17 (14.41) |  |
| Unmarried | | 21 (9.72) |  | 5 (5.10) | 16 (13.56) |  |
| ^c^ Two-sample t-tests or Chi-square-tests  ^d^ No summation to 100% due to multiple responses | | | | | | |
|  |  |  |  |  |  |  |

| **Supplementary Table 2.** Mean person measures considering different modes of administration | | | |
| --- | --- | --- | --- |
|  | **IVI_F** | **IVI_E** |  |
| First phone interview | 1.86 | 1.71 |  |
| Second phone interview | 1.56 | 1.76 |  |
| Paper | 1.31 | 1.32 |  |
| Electronic | 1.76 | 1.60 |  |

IVI_F, functional subscale; IVI_E, emotional subscale

| **Supplementary Table 3.** Analysis investigating mean test-retest differences of IVI person measures by self-reported hearing difficulties, initial mode of administration, administration interval and best-corrected visual acuity | | | | | |
| --- | --- | --- | --- | --- | --- |
| **IVI_F** |  |  | |  |  |
| Phone-Paper |  |  | Mean difference ± SD | p-value | corrected p-value |
|  | Self-reported hearing difficulties | No | 0.60 ±1.10 |  |  |
|  |  | Yes | 0.58 ±0.84 | 0.869 | 1.0 |
|  | Inital mode of administration | phone | 0.54 ±1.07 |  |  |
|  |  | paper | 0.67 ±1.04 | 0.130 | 1.0 |
|  | Administration interval | ≤2 weeks | 0.59 ±1.07 |  |  |
|  |  | >2 weeks | 0.69 ±1.01 | 0.812 | 1.0 |
|  | Best-corrected visual acuity | logMAR <0.3 | 0.68 ±1.11 |  |  |
|  |  | logMAR ≥0.3 | 0.42 ±0.85 | 0.039 | 0.702 |
|  | Psychiatric diseases | No | 0.65 ±1.02 |  |  |
|  |  | Yes | 0.42 ±1.19 | 0.525 | 1.0 |
| Retest | Self-reported hearing difficulties | No | 0.29 ±0.85 |  |  |
|  |  | Yes | 0.19 ±0.59 | 0.363 | 1.0 |
|  | Administration interval | ≤2 weeks | 0.23 ±0.76 |  |  |
|  |  | >2 weeks | 0.31 ±0.82 | 0.199 | 1.0 |
|  | Best-corrected visual acuity | logMAR <0.3 | 0.34 ±0.85 |  |  |
|  |  | logMAR ≥0.3 | 0.11 ±0.61 | 0.059 | 1.0 |
|  | Psychiatric diseases | No | 0.30 ±0.77 |  |  |
|  |  | Yes | 0.10 ±0.90 | 0.320 | 1.0 |
| **IVI_E** |  |  | |  |  |
| Phone-Paper |  |  | Mean difference ± SD | p-value | corrected p-value |
|  | Self-reported hearing difficulties | No | 0.36 ±0.93 |  |  |
|  |  | Yes | 0.51 ±0.78 | 0.150 | 1.0 |
|  | Inital mode of administration | phone | 0.38 ±0.94 |  |  |
|  |  | paper | 0.43 ±0.85 | 0.369 | 1.0 |
|  | Administration interval | ≤2 weeks | 0.41 ±0.81 |  |  |
|  |  | >2 weeks | 0.39 ±1.12 | 0.975 | 1.0 |
|  | Best-corrected visual acuity | logMAR <0.3 | 0.42 ±0.92 |  |  |
|  |  | logMAR ≥0.3 | 0.36 ±0.82 | 0.781 | 1.0 |
|  | Psychiatric diseases | No | 0.44 ±0.89 |  |  |
|  |  | Yes | 0.26 ±0.87 | 0.315 | 1.0 |
| Retest | Self-reported hearing difficulties | No | -0.05 ±0.90 |  |  |
|  |  | Yes | -0.07 ±0.91 | 0.634 | 1.0 |
|  | Administration interval | ≤2 weeks | -0.07 ±0.92 |  |  |
|  |  | >2 weeks | -0.05 ±0.89 | 0.882 | 1.0 |
|  | Best-corrected visual acuity | logMAR <0.3 | -0.06 ±0.85 |  |  |
|  |  | logMAR ≥0.3 | -0.06 ±1.03 | 0.985 | 1.0 |
|  | Psychiatric diseases | No | -0.05 ±0.91 |  |  |
|  |  | Yes | -0.10 ±0.88 | 0.907 | 1.0 |

IVI_F, functional subscale; IVI_E, emotional subscale; SD, standard deviation

**Supplementary Figure 1.** IVI score test-retest differences by visual acuity


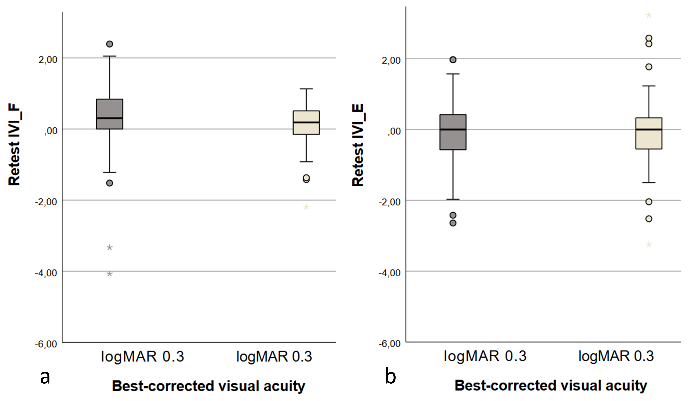


**Supplementary Figure 2.** IVI score test-retest differences by administration interval


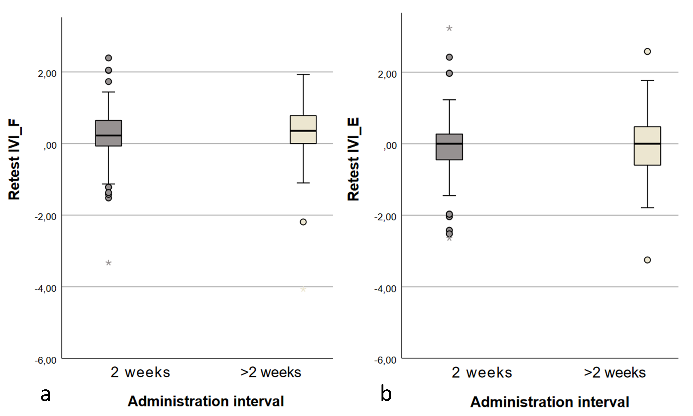


**Supplementary Figure 3.** IVI score test-retest differences by self-reported hearing difficulties


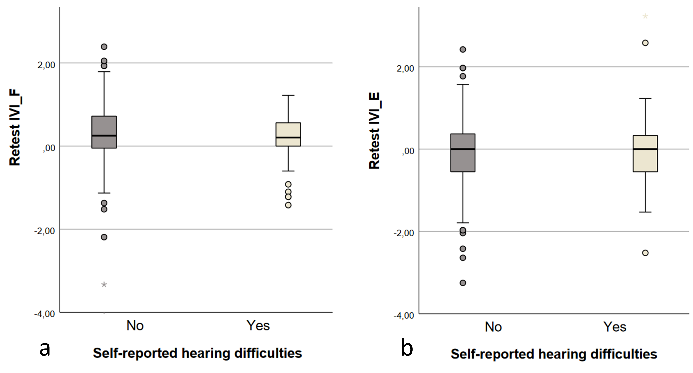


**Supplementary Figure 4.** IVI score test-retest differences by self-reported psychiatric diseases


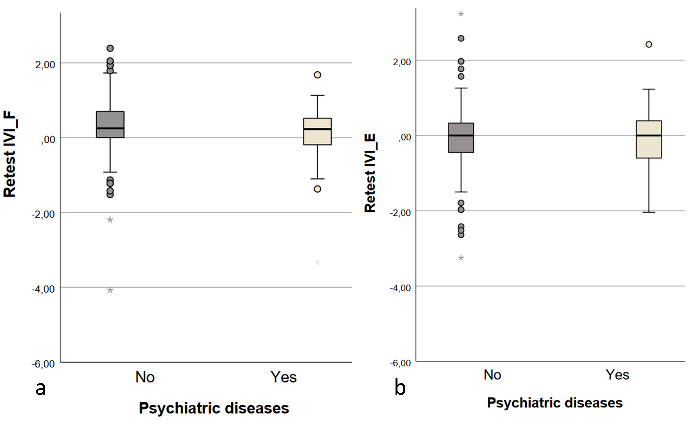


**Supplementary Figure 5.** IVI score inter-mode differences by visual acuity


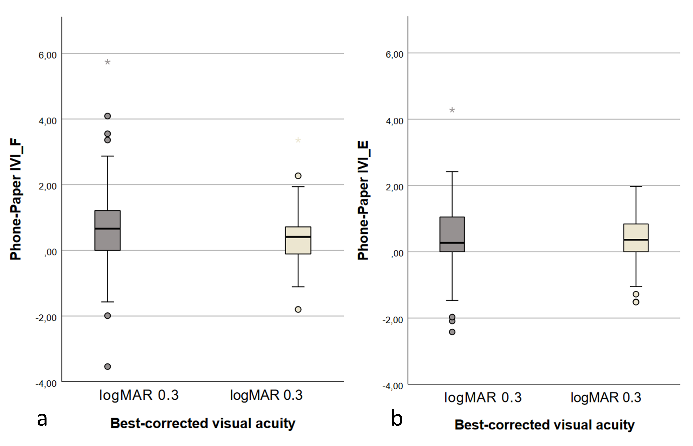


**Supplementary Figure 6.** IVI score inter-mode differences by administration interval


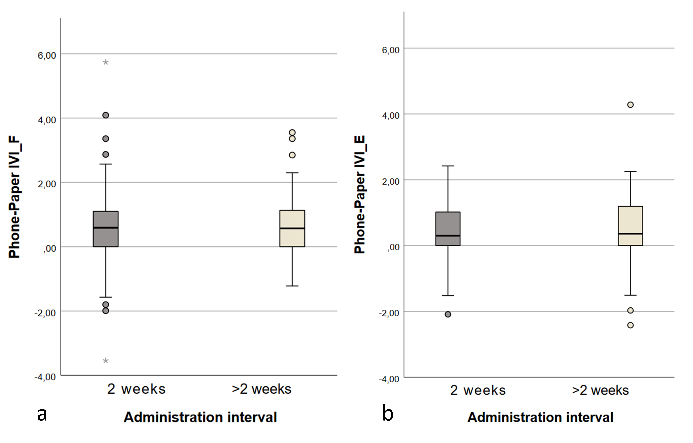


**Supplementary Figure 7.** IVI score inter-mode differences by initial mode of administration


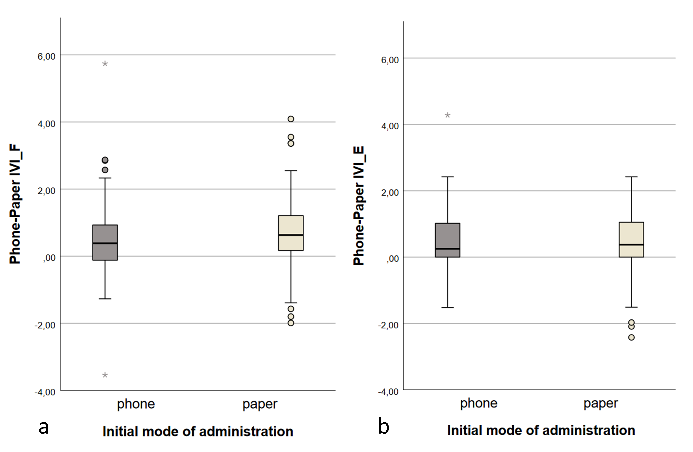


**Supplementary Figure 8.** IVI score inter-mode differences by self-reported hearing difficulties


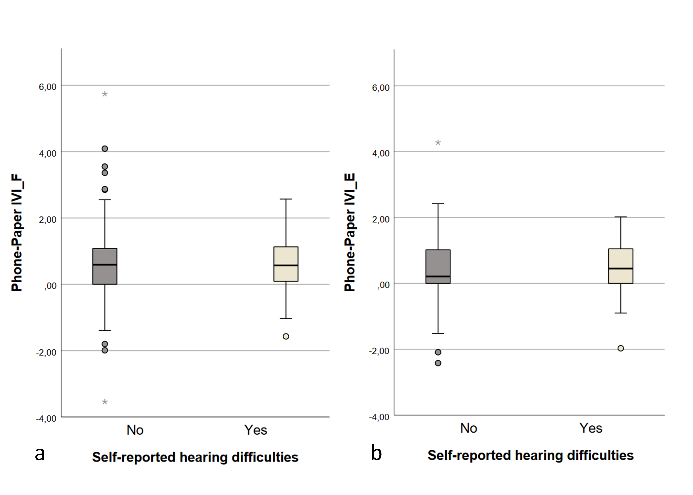


**Supplementary Figure 9.** IVI score inter-mode differences by self-reported psychiatric diseases


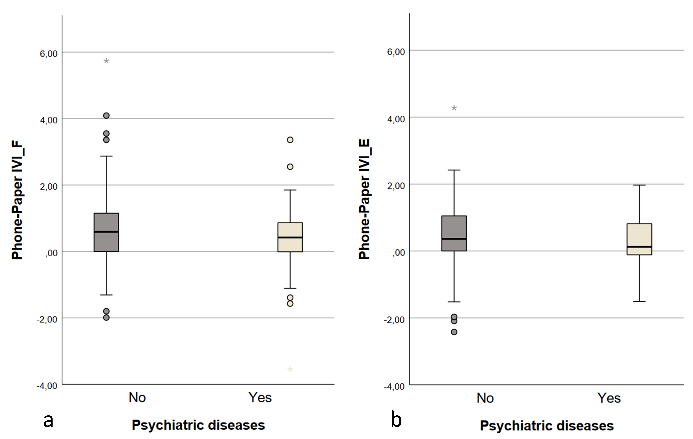

Supplement: Supplementary file 1 — Supplementary file1 (DOCX 645 KB) [file 417_2023_6334_MOESM1_ESM.docx]
